# FAM83A promotes proliferation and metastasis via Wnt/β-catenin signaling in head neck squamous cell carcinoma

**DOI:** 10.1186/s12967-021-03089-6

**Published:** 2021-10-12

**Authors:** Huan Ji, Haiyang Song, Zeyu Wang, Pengfei Jiao, Jiani Xu, Xuan Li, Hongming Du, Heming Wu, Yi Zhong

**Affiliations:** 1 Jiangsu Province Key Laboratory of Oral Diseases, School of Stomatology, Nanjing Medical University, Nanjing, China; 2 Jiangsu Province Engineering Research Center of Stomatological Translational Medicine, School of Stomatology, Nanjing Medical University, Nanjing, China; 3 Department of Oral and Maxillofacial Surgery, The Affiliated Stomatological Hospital of Nanjing Medical University, Nanjing, China; 4 Department of General Dentistry, Department of Oral Medicine, The Affiliated Stomatological Hospital of Nanjing Medical University, #136 Hanzhong Road, Nanjing, 210029 Jiangsu China

**Keywords:** FAM83A, Head Neck Squamous Cell Carcinoma, Wnt/β-catenin Signaling, Proliferation, Metastasis

## Abstract

This research aimed to investigate the expression and function of FAM83A in the proliferation and metastasis in head and neck squamous cell carcinoma (HNSCC). FAM83A mRNA and protein expressions in HNSCC were detected in primary HNSCC samples and cell lines. The associations between FAM83A expression and clinicopathologic variables were evaluated through tissue microarrays. Besides, FAM83A knockdown and overexpression cell lines were constructed to assess cell growth and metastasis in vitro and the relationship between FAM83A and epithelial-mesenchymal transition (EMT). Furthermore, two models of xenograft tumors in nude mice were used to assess the tumorigenicity and metastasis ability of FAM83A in vivo. In the present study, overexpression of FAM83A in HNSCC samples was significantly associated with tumor size, lymph node status and clinical tumor stages. Mechanically, FAM83A could promote HNSCC cell growth and metastasis by inducing EMT via activating Wnt/β-catenin signaling pathway. Rescue experiment demonstrated the inhibition of β-catenin could counteract the function of FAM83A. Also, the FAM83A knockdown could suppress tumor growth and distant metastasis in the xenograft animal models of HNSCC. In conclusion, this study identifies FAM83A as an oncogene of HNSCC. This study provides new insights into the molecular pathways that contribute to EMT in HNSCC. We revealed a previously unknown FAM83A-Wnt–β-catenin signaling axis involved in the EMT of HNSCC. There may be a potential bi-directional signaling loop between FAM83A and Wnt/β-catenin signaling pathway in HNSCC.

**Supplementary Information:**

The online version contains supplementary material available at 10.1186/s12967-021-03089-6.

## Introduction

In recent years, head and neck squamous cell carcinoma (HNSCC) is one of the most common cancers worldwide, with more than 350,000 cancer-related deaths per year even though combined and multidisciplinary therapy has greatly advanced [1, 2]. Therefore, more efforts should be made to explore the cellular and molecular mechanism of HNSCC tumorigenesis. Thus, the dentification of new therapeutic targets for HNSCC is urgently needed for clinicians.

As the smallest member of the FAM83 family (FAM83A to H), family with sequence similarity 83, member A (FAM83A), is located on chromosome 8q24. In 2005, FAM83A with the conserved DUF1669 domain at its N-terminus, was firstly reported as a potential cancer biomarker involved in tumor progression [3]. Prior studies have suggested that FAM83A is abnormally expressed and involved in the progression of multiple human cancers [4–13]. It is also reported FAM83A can be a diagnostic and prognostic marker of non-small cell lung cancer and is closely related to tumor histology and signal transduction [14, 15]. FAM83A signaling can also stimulate epithelial-mesenchymal transition (EMT) by triggering the PI3K/AKT/Snail pathway in non-small cell lung cancer [16]. Furthermore, FAM83A/PD-L1 co-expression correlates with poor prognosis in lung adenocarcinoma, and FAM83A drives PD-L1 expression via ERK signaling, thus causing tumor immune escape [13].

DUF1669 domain mediates the interaction with the α, δ or ε isoforms of the CK1 family of Ser/Thr protein kinases [17]. The FAM83 proteins can interact with CK1α, and FAM83A, B, E and H also interact withCK1δ and ε isoforms.CK1α, δ and ε isoforms have been involved in cellular processes including Wnt signaling, mitosis, and DNA damage responses [18–21].It has been previously reported that FAM83F and FAM83G can regulate canonical Wnt signaling through an interaction with CK1α [22]. Recent studies have shown that FAM83A also regulates canonical Wnt/β-catenin signaling pathway in EMT of lung cancer [23]. However, whether FAM83A can regulate canonical Wnt signaling in HNSCC remains unknown. Here, we aim to clarify the role and mechanism of FAM83A in the proliferation and metastasis of HNSCC cells, thus providing evidence for more potential therapeutic targets for HNSCC.

## Materials and methods

### Patients

In brief, 242 patients including 148 males and 94 females, who underwent surgery and were diagnosed as primary HNSCC from 2009 to 2014 in the Affiliated Stomatological Hospital of Nanjing Medical University, were recruited. 242 HNSCC samples and 12 HNSCC adjacent normal tissues were used to make tissue microarrays [24]. The clinicopathologic information including age, gender, tumor size, lymph node status, histological grade, clinical stage and follow-up information were obtained from the patients’ electronic medical records and follow-up visits. Besides, we collected 48 pairs of fresh HNSCC tissues and adjacent normal tissues to extract mRNA and chose 10 pairs of them to examine FAM83A expression. All experiments were in accordance with the Institutional Review Board of the Nanjing Medical University and complied with the Declaration of Helsinki (Approval ID 2019343).

### Immunohistochemistry and evaluation of immunoreactivity

The procedure of immunohistochemistry (IHC) was carried out as we described previously [25]. The stained slides were analyzed by two pathologists separately. FAM83A staining was grouped by combining intensity score (IS) and positive score (PS). IS was divided into four groups: negative (0), weak (1), moderate (2), and strong (3), whereas PS was categorized into four grades: negative (0), < 10% (1), 11%-50% (2), 51%-80% (3), and > 80% (4). We then calculated the immunoreactive score (IRS) by multiplying IS and PS. The final IRSs were divided into two groups: low FAM83A expression (≤ 4), and high FAM83A expression (> 4). The antibody used in IHC was FAM83A (1:100; Proteintech, Rosemont, IL, USA).

### Cell lines and cell culture

The human normal oral keratinocytes (HOK) and the human HNSCC cell lines including CAL27, FADU, HN4, HN6 SCC-9, and SCC-25 cell, were purchased from China Center for Type Culture Collection (Shanghai, China), CAL27 and HOK were cultured in DMEM medium (Gibco). FADU, HN4, and HN6 were cultured in DMEM/F12 medium (Gibco). All culture medium contained 10% fetal bovine serum (FBS, Sciencell) and 1% penicillin/streptomycin. Cells were cultured in a humidified incubator containing 5% CO2 at 37 °C.

### Cell transfection

The human FAM83A lentivirus targeting to upregulate FAM83A (LV-FAM83A), the negative control (LV-NC), the shRNA lentivirus targeting to knock down FAM83A (shFAM83A: 5’-GGAGUGUGGAAGGAGAGAUTT-3’), and the shRNA negative control lentivirus (shNC) were ordered from GeneChem Co., Ltd (Shanghai, China). Small interfere RNA (siRNA) of β-catenin (siβ-catenin: 5’-GGACACAGCAGCAAUUUGUTT-3’) purchased from GenePharma Co., Ltd (Shanghai, China) was used to silence β-catenin expression. A plasmid containing the β-catenin cDNA sequence (GeneCoperia, China) was used to overexpress β-catenin expression. XAV-939 (Selleck, USA), a small-molecule inhibitor, resolved with DMSO, was used to suppress exogenous β-catenin expression.

### Real-time PCR and western blotting assays

Real-time RT-PCR and western blotting assays were carried out in accordance with standard process as we described previously [25]. Nuclear and cytoplasmic protein extraction from cells is in accordance with the TRIZOL reagent (Invitrogen) manufacturer’s protocol. The primers were used as follows, FAM83A: forward 5’- ATCCGGAGTGTGGAAGGAGAG -3’, reverse 5’- TCCAGACAGGACAAATCTCCAGT -3’; E-cadherin: forward 5’-GCCTTATGATTCTCTGCTCGTG -3’, reverse 5’-GCCCCATTCGTTCAAGTAGTC-3’; N-cadherin: forward 5’-GTGAGCCTGCAGATTTTAAGGTG-3’, reverse 5’-GTTGGCTTCAGGCTCATTTTACT-3’; Vimentin: forward 5’-CTGGATTCACTCCCTCTGGTT-3’, reverse 5’-TCGTGATGCTGAGAAGTTTCGTT-3’; Snail: forward 5’-TTCTCACTGCCATGGAATTCC-3’, reverse 5’- GCAGAGGACACAGAACCAGAAA-3’; β-catenin: forward 5’- TGACAAAACTGCTAAATGACGAGG-3’, reverse 5’- CGCATGATAGCGTGTCTGGA-3’; c-myc: forward 5’- CCACGAAACTTTGCCCATAG -3’, reverse 5’- TGCAAGGAGAGCCTTTCAGAG-3’;Cyclin D1: forward 5’- TGTCCCACTCCTACGATACGC -3’, reverse 5’- CAGCATCTCATAAACAGGTCACTAC-3’; GAPDH: forward 5’-GACGTAGGGAGTGAAGGT C-3’, reverse 5’-GAGAGTTCAGATGTTGATGG-3’. Primary antibodies were as follows: GAPDH (1:1000; Proteintech, Rosemont, IL, USA), FAM83A (1:1000; Proteintech, Rosemont, IL, USA), E-cadherin (1:1000; CST), N-cadherin(1:1000; CST), Vimentin (1:1000; CST), Snail (1:1000; Proteintech, Rosemont, IL, USA), β-catenin (1:1000; CST), Phospho-β-catenin (1:1000; CST), Lamin B1 (1:1000; CST), c-myc (1:1000; CST), and Cyclin D1 (1:1000; CST).

### Cell migration and invasion assays

For the migration assay, 8 × 10^4^ HNSCC cells in 200ul complete medium were seeded into the upper compartment of a transwell insert with 8 µm pores (Costar, Lowell, MA, USA). The lower chamber was filled with 700ul basal medium containing 10% fetal bovine serum (FBS). After 12 h (HN6 cell line) or 24 h (CAL27, FADU and HN4 cell lines), the invaded cells adhering to the lower compartment were fixed, stained, and counted under an inverted microscope (Olympus, Tokyo, Japan). For the invasion assay, 2 × 10^5^ cells/well were plated and the upper compartment was pre-coated with Matrigel (Corning, Bedford, MA, USA).

### Cell viability measurement

Cell viability was detected by CCK-8 assay (Dojindo Molecular Technologies, Kumamoto, Japan). Cells were seeded and cultured in 96-well plates at a density of 2000 cells/well. Every other day, CCK-8 reagent was added and the absorbance was determined at 450 nm.

### Wound-healing assay

Cells were seeded into six-well plates and scratched with a 10ul pipette tip when cells grew to 90% confluence, and then cells were incubated with complete medium. At 0, 12, or 24 h, wound closure was photographed and its percentage was calculated.

### Immunofluorescence staining

Cells were plated onto coverslips and fixed in 4% paraformaldehyde for 20 min when cells grew to 50% confluency. Then, the coverslips were permeabilized with or without 0.3% Triton, incubated with goat serum for 30 min and primary antibody overnight at 4 ℃. After it was rinsed thrice with PBS with Tween-20, cells were.

incubated with fluorescent Cy3 secondary antibodies (1:50, Proteintech, USA) for 1 h at 37 °C in the dark. After the slides were incubated with DAPI (Life Technologies, USA), cells were observed and photographed under an FV1000 laser confocal scanning microscope (Tokyo, Japan). Primary antibodies used were as follows: E-cadherin (1:100, CST), Vimentin (1:100, CST), and β-catenin (1:100; CST).

### In vivo assay

All animal experiments were in accordance with the Animal Use and Care Committee of the Affiliated Hospital of Stomatology, Nanjing Medical University (IACUC-1906018). Female 4–6 weeks old BALB/c nude mice used were purchased from Vital River Laboratory Animal Technology Co.Ltd (Beijing, China). To explore the role of FAM83A on HNSCC tumor growth, a total of 2 × 10^6^ FAM83A knockdown HN6 cells and control cells were injected into the right armpit of mice. Tumor growth and body weight were determined every three days. After 24 days, mice were killed and tumors were stripped carefully. To explore the role of FAM83A on HNSCC tumor metastatic potential, a total of 1 × 10^6^ HN6 cells were injected into the nude mice by tail intravenous. After 42 days, mice lungs were dissected carefully and the number of metastatic nodes were counted.

### Statistical analysis

The chi-square test, Fisher’s exact probability method, Mann–Whitney U test and Kruskal–Wallis test were applied to analyze the connection between FAM83A expression and clinicopathological characteristics. The Kolmogorov–Smirnov test and paired-samples T test were applied to compare FAM83A mRNA expression in HNSCC samples and adjacent normal tissues. Statistical significance in in vitro and in vivo experiments was assessed using one-way ANOVA and independent-samples T test. Data were presented as the mean ± SD from three independent experiments and all data were analyzed via GraphPad Prism 7 (San Diego, CA, USA) software. *P* < 0.05 was considered significant in all experiments.

## Results

### FAM83A overexpression was associated with clinicopathological characteristics in HNSCC

**Fig. 1:**
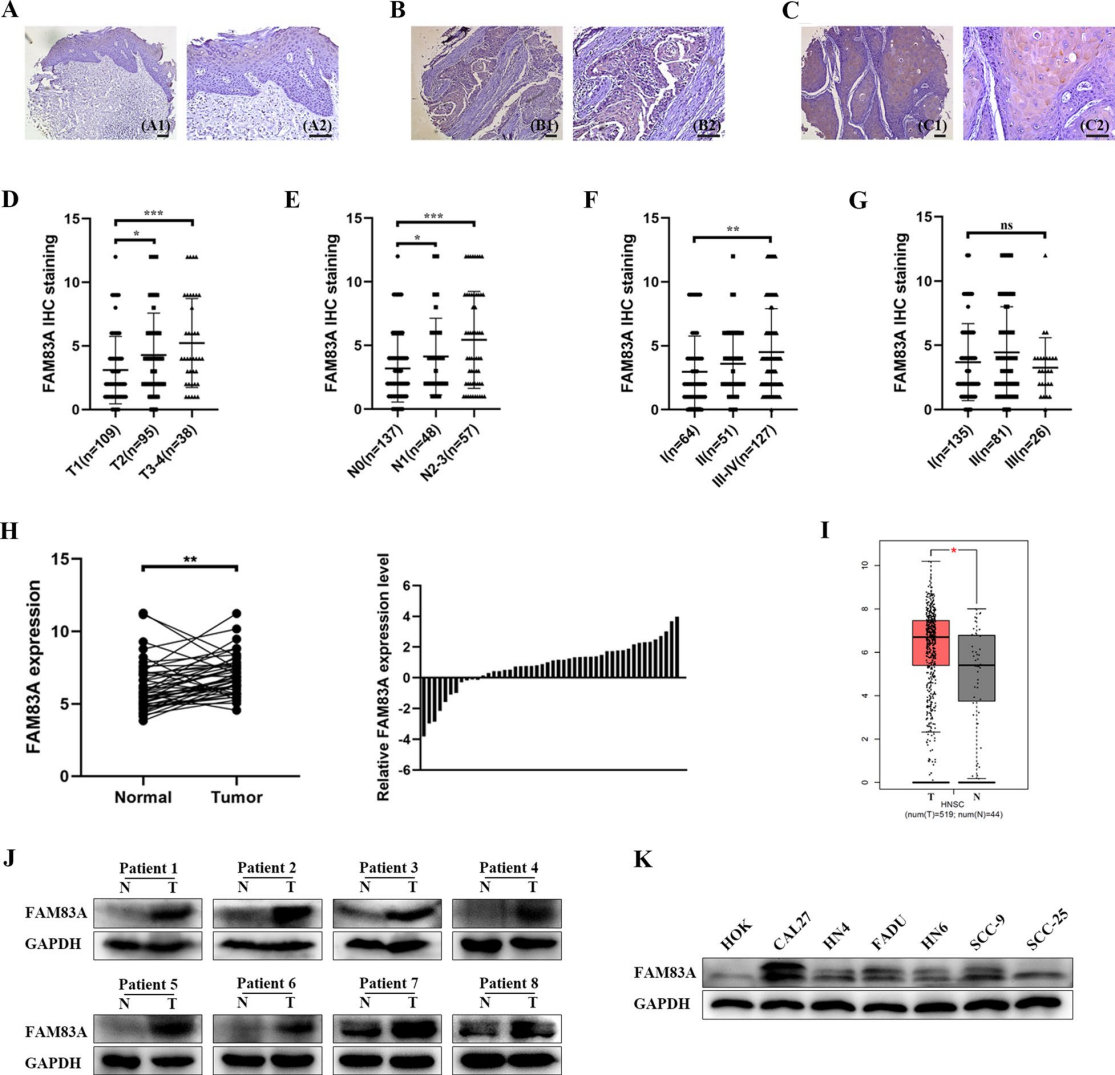
FAM83A is overexpressed and related to tumor size, lymph node metastasis and clinical tumor stages in HNSCC. **A** Negative FAM83A expression in HNSCC adjacent normal tissues (A1, 100 × ; A2, 200 ×). **B** Low FAM83A expression in HNSCC tumor tissues (B1, 100 × ; B2, 200 ×). **C** High FAM83A expression in HNSCC tumor tissues (C1, 100 × ; C2, 200 ×). **D** Quantification analysis of FAM83A staining in HNSCC with different tumor size in microarrays. **E** Quantification analysis of FAM83A staining in HNSCC with different lymph node metastasis in microarrays. **F** Quantification analysis of FAM83A staining in HNSCC with different clinical tumor stages in microarrays. **G** Quantification analysis of FAM83A staining in HNSCC with different pathological classifications in microarrays. **H** The relative expression of FAM83A mRNA was measured by qRT-PCR in 48 freshly collected HNSCC samples and paired adjacent non-tumor tissues. The height of column represents the fold change (log2-transformed) in FAM83A expression in these 48 patients (right panel). **I** Differential expressions of FAM83A in HNSCC (n = 519) and normal tissues (n = 44) from the TCGA database. **J** The expressions of FAM83A protein in eight HNSCC tissue and matched adjacent normal tissues. **K** The expressions of FAM83A protein in HOK and HNSCC cell lines. *p < 0.05, **p < 0.01, ***p < 0.001

To explore the role of FAM83A in HNSCC, we examined FAM83A expression through 3 tissue microarrays consisting of 242 human HNSCC samples and 12 adjacent normal tissues through IHC analysis. The expression patterns of FAM83A in HNSCC and adjacent normal tissues were classified according to immunoreactive scores and the detailed demographic and clinicopathological parameters of these patients were listed in Table 1. Obviously, HNSCC tissues tended to show stronger cytoplasmic staining of FAM83A and adjacent normal tissues exhibited a lower or negative expression of FAM83A (Fig. 1A–C). We then analyzed the relationship between FAM83A expression and clinicopathological features (Table 1 and Fig. 1D–G). Elevated expressions of FAM83A were detected in tumor samples with bigger tumor size and higher clinical tumor stages (Fig. 1D, F). Besides, advanced HNSCC patients with lymph node metastasis showed higher FAM83A expression levels than those in early-stage patients with no lymph node metastasis (Fig. 1E). However, FAM83A expression has no correction with pathological classifications (Fig. 1G). Above all, we found that increased FAM83A expression was related to tumor volumes, lymph node status, and clinical tumor stages.

**Table 1:** Correlation between FAM83A expression and multiple clinicopathological parameters in HNSCC

Pathologic characteristics	n	FAM83A expression (number of cases)		*P* value
		Low	High	
Age, years				
≥ 60	118	88	30	0.2412
< 60	124	84	40	
Sex				
Male	148	115	33	0.0056
Female	94	57	37	
Tumor size				
T1	109	87	22	0.0399
T2	95	63	32	
T3	24	14	10	
T4	14	8	6	
Lymph node status				
N0	130	108	22	< 0.0001
N1	41	31	10	
N2	27	17	10	
N3	44	16	28	
Pathological grade				
I	136	99	37	0.2984
II	81	53	28	
III	25	20	5	
Clinical stage				
I	64	52	12	0.0983
II	51	36	15	
III	35	26	9	
IV	92	58	34

To further clarify the role of FAM83A in HNSCC, we collected 48 pairs of fresh tumor tissues which included HNSCC and adjacent normal tissues. Forty-eight pairs of tissues were used to extract mRNA and 10 pairs of tissues among them were used to extract protein. The qRT-PCR results in 48 HNSCC samples and paired normal tissues demonstrated that FAM83A mRNA was significantly increased in HNSCC than paired normal tissues (Fig. 1H). Among 10 pairs of HNSCC samples and normal tissues, 8 pairs presented higher FAM83A protein levels in tumor tissues (Fig. 1J). FAM83A presented a higher expression level in HNSCC samples than adjacent normal tissues both at mRNA and protein level. Besides, TCGA database showed that the FAM83A mRNA expression is markedly upregulated in 519 HNSCC samples compared to 44 normal tissues (Fig. 1I). Meanwhile, we found that FAM83A expressions in HNSCC cell lines were higher than that in human normal oral keratinocytes (HOK) (Fig. 1K).

### FAM83A in HNSCC promoted tumor growth and metastasis in vitro

**Fig. 2:**
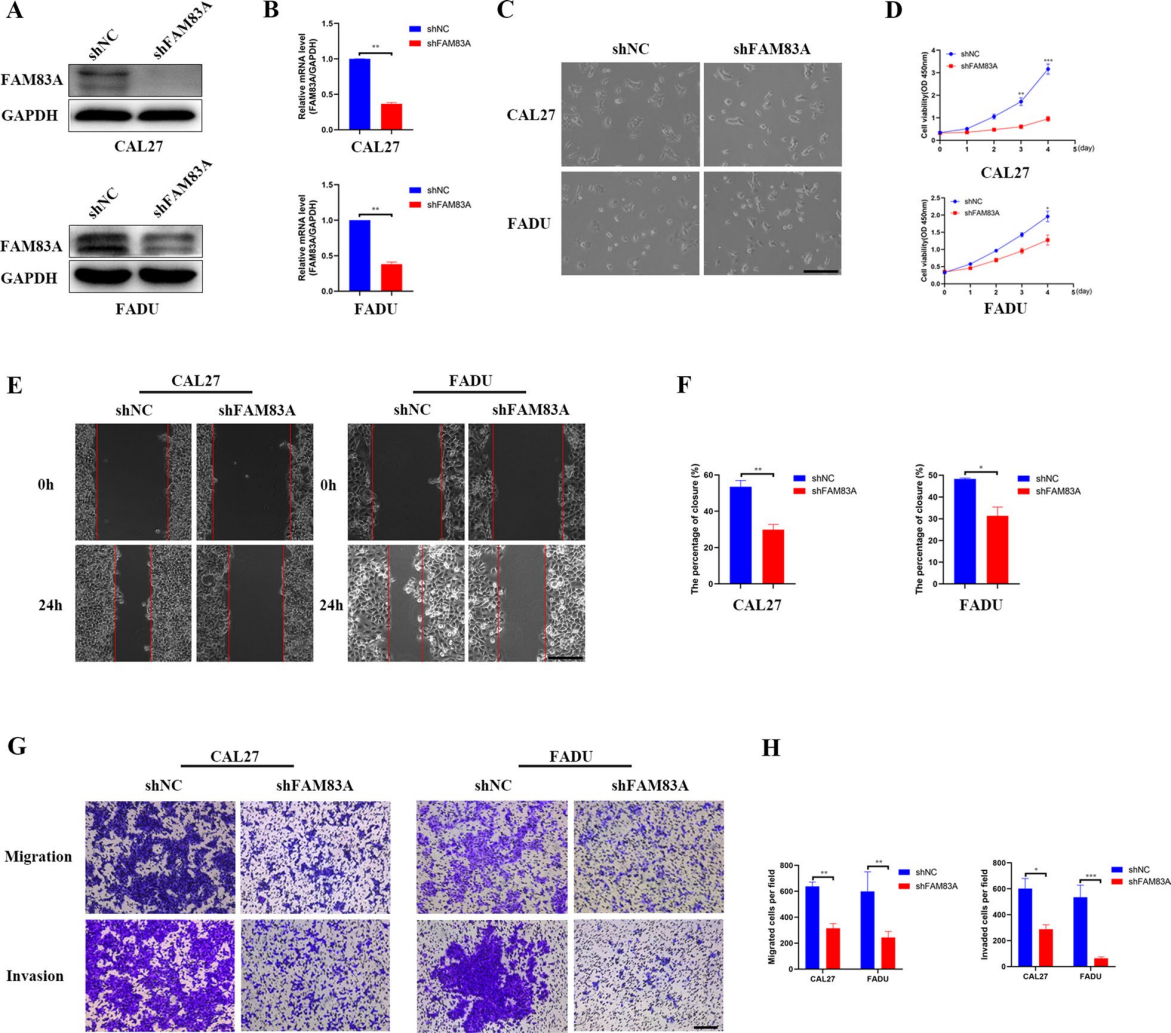
FAM83A knockdown in CAL27 and FADU cells inhibits cell growth and metastasis. **A**, **B** FAM83A expression based on real-time PCR and western blotting in CAL27 and FADU cell lines after shFAM83A transfection. **C** Morphological changes of Cal27 and FADU cells after shFAM83A transfection (100 ×). **D** CCK8 assays of the proliferation in CAL27 and FADU cells after shFAM83A transfection. **E**, **F** The wound-healing assays in CAL27 and FADU cells after shFAM83A transfection (100 ×). **G**, **H** Transwell assays of migration and invasion in CAL27 and FADU cells after shFAM83A transfection (100 ×). Data represent the mean ± SD; *p < 0.05, **p < 0.01, ***p < 0.001

**Fig. 3:**
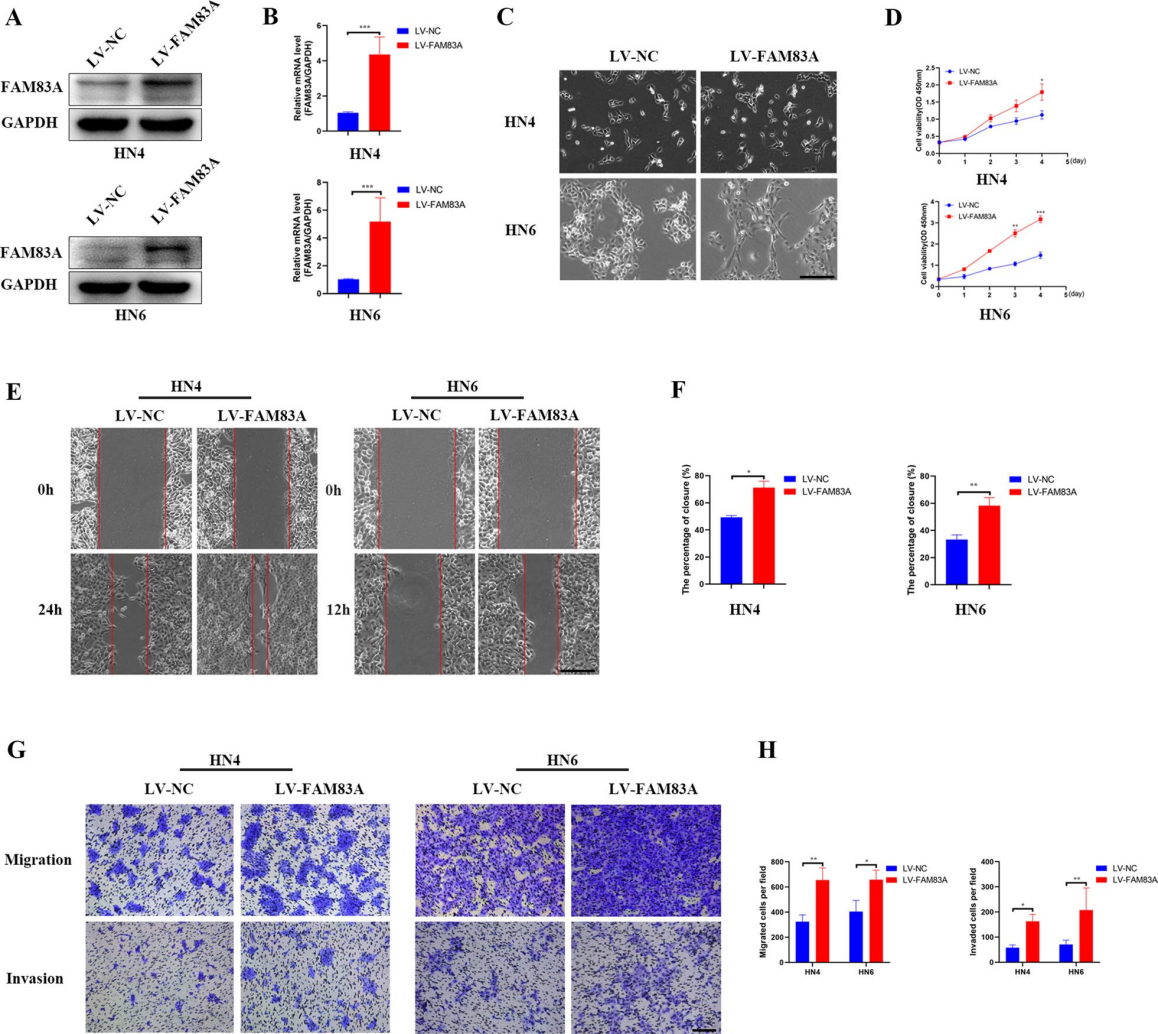
FAM83A overexpression in HN4 and HN6 cells promotes cell growth and metastasis. **A**, **B** FAM83A expressions based on real-time PCR and western blotting in HN4 and HN6 cell lines after treated with LV-FAM83A. **C** Morphological changes of HN4 and HN6 cells after LV-FAM83A transfection (100 ×).** D** CCK8 assays of the proliferation in HN4 and HN6 cells after LV-FAM83A transfection. **E**, **F** A wound-healing assay in HN4 and HN6 cells after LV-FAM83A transfection (100 ×). **G**, **H** Transwell assays of migration and invasion in HN4 and HN6 cells after LV-FAM83A transfection (100 ×). Data represent the mean ± SD; *p < 0.05, **p < 0.01, ***p < 0.001

To further explore the function of FAM83A in HNSCC, we constructed the FAM83A knockdown and overexpression cell lines and verified the efficiency of interference both at the mRNA and protein levels through real-time PCR and western blotting assays. As shown in Fig. 1K, the relatively endogenous FAM83A in CAL27 and FADU cells was higher, and FAM83A in HN4 and HN6 cells was lower. Herein, CAL27 and FADU cells were chosen to be transfected with specific shRNA lentivirus (Fig. 2A, B), while HN4 and HN6 cells were transfected with FAM83A overexpression lentivirus (LV-FAM83A) (Fig. 3A and B). Interestingly, we found that FAM83A knockdown did not change CAL27 and FADU cell morphology (Fig. 2C), but FAM83A overexpression made HN4 and HN6 cells become elongated and fibroblast-like (Fig. 3C). Meanwhile, CCK8 assay found FAM83A downregulation impaired cell viability in CAL27 and FADU cells (Fig. 2D), while, FAM83A upregulation promoted cell growth in HN4 and HN6 cells (Fig. 3D). In the wound healing experiment, the migration of FAM83A knockdown group was significantly slower than those of the negative control group in CAL27 and FADU cell lines (Fig. 2E, F). Conversely, FAM83A overexpression group enhanced the migratory ability in HN4 andHN6 cells lines (Fig. 3E, F). Besides, transwell assays showed that the downregulation of FAM83A significantly weakened the migratory and invasive ability in CAL27 and FADU cell lines (Fig. 2G, H) and FAM83A upregulation significantly enhanced the migratory and invasive ability in HN4 and HN6 cell lines (Fig. 3G and H). These results indicated FAM83A promoted HNSCC cells growth and metastasis in vitro.

### FAM83A promoted EMT and activated Wnt/β-catenin signaling pathway in HNSCC cells

**Fig. 4:**
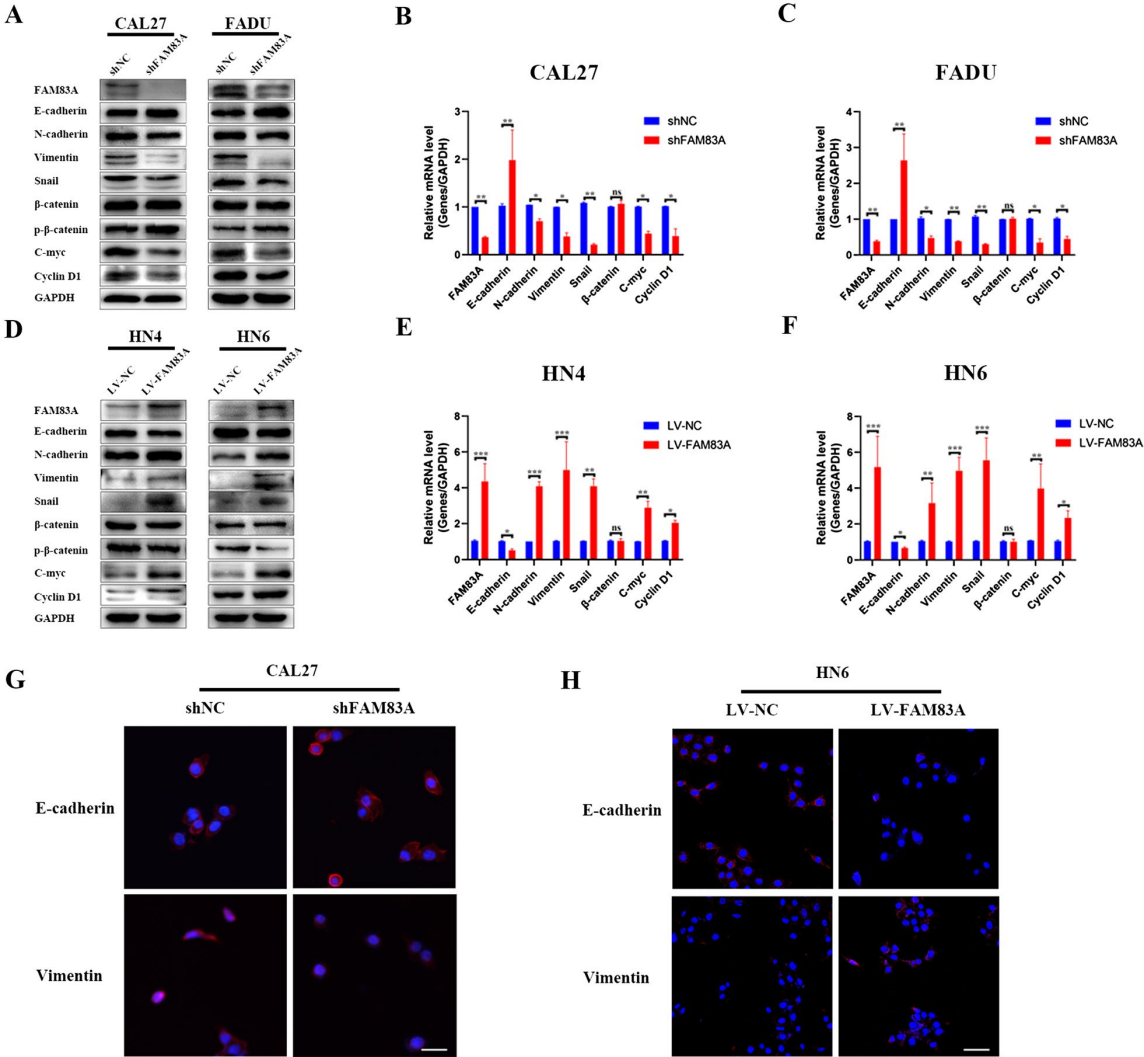
FAM83A promotes EMT and activates Wnt/β-catenin signaling pathwayin HNSCC cells. **A** Protein levels of FAM83A, E-cadherin, N-cadherin, Vimentin, Snail and Wnt-responsive genes including β-catenin, phosphor-β-catenin, c-myc and Cyclin D1 were determined by western blotting in CAL27 and FADU cells after shFAM83A transfection. **B**, **C** Gene expressions of FAM83A, E-cadherin, N-cadherin, Vimentin, Snail, c-myc and Cyclin D1 were measured by RT-PCR in CAL27 and FAUD cells after shFAM83A transfection. **D** Protein levels of FAM83A, E-cadherin, N-cadherin, Vimentin, Snail and Wnt-responsive gene including β-catenin, phosphor-β-catenin, c-myc and Cyclin D1 were determined by western blotting in HN4 and HN6 cells after LV-FAM83A transfection. **E**, **F** Gene expressions of FAM83A, E-cadherin, N-cadherin, Vimentin, Snail and c-myc and Cyclin D1 were measured by RT-PCR in HN4 and HN6 cells after LV-FAM83A transfection. **G** Immunofluorescence analysis of the EMT marker E-cadherin and Vimentin in CAL27 cells after shFAM83A transfection (200 ×). **H** Immunofluorescence analysis of the EMT marker E-cadherin and Vimentin in HN6 cells after LV-FAM83A transfection (200 ×). Data represent the mean ± SD; *p < 0.05, **p < 0.01, ***p < 0.001

The morphological and functional results induced by FAM83A suggested FAM83A may be an important facilitator of EMT in HNSCC. Herein, we determined EMT-related markers expression through western blotting, real-time PCR and immunofluorescence assays after FAM83A silencing and overexpression. As shown in Fig. 4A–H, after FAM83A was knocked down in CAL27 and FADU cells, the epithelial marker E-cadherin was elevated and the mesenchymal markers including N-cadherin, Vimentin and Snail descended at both protein and mRNA levels (Fig. 4A–C). As expected, FAM83A overexpression exerted the opposite effects in HN4 and HN6 cells (Fig. 4D–F). Both real-time PCR and western blotting analysis validated this result. Furthermore, immunofluorescence staining assay confirmed the downregulation of FAM83A decreased Vimentin but increased E-cadherin expression in CAL27 cell lines, whereas the upregulation of FAM83A reverted this phenomenon in HN6 cell lines (Fig. 4G, H). Besides, we found FAM83A could enhance Wnt-responsive genes expression. As shown in Fig. 4A–F, FAM83A knockdown group cells showed the downregulation of c-myc and Cyclin D1 and the upregulation of phosphor-β-catenin. Meanwhile, FAM83A overexpression group cells showed the upregulation of c-myc and Cyclin D1 and the downregulation of phosphor-β-catenin compared to the negative control group. However, FAM83A did not affect total β-catenin expression in HNSCC cells (Fig. 4A–F). Taken together, FAM83A promoted EMT and activated Wnt/β-catenin signaling pathway in HNSCC cell lines.

### FAM83A promoted EMT through Wnt/β-catenin signaling pathway and β-catenin regulated FAM83A expression in HNSCC cells

**Fig. 5:**
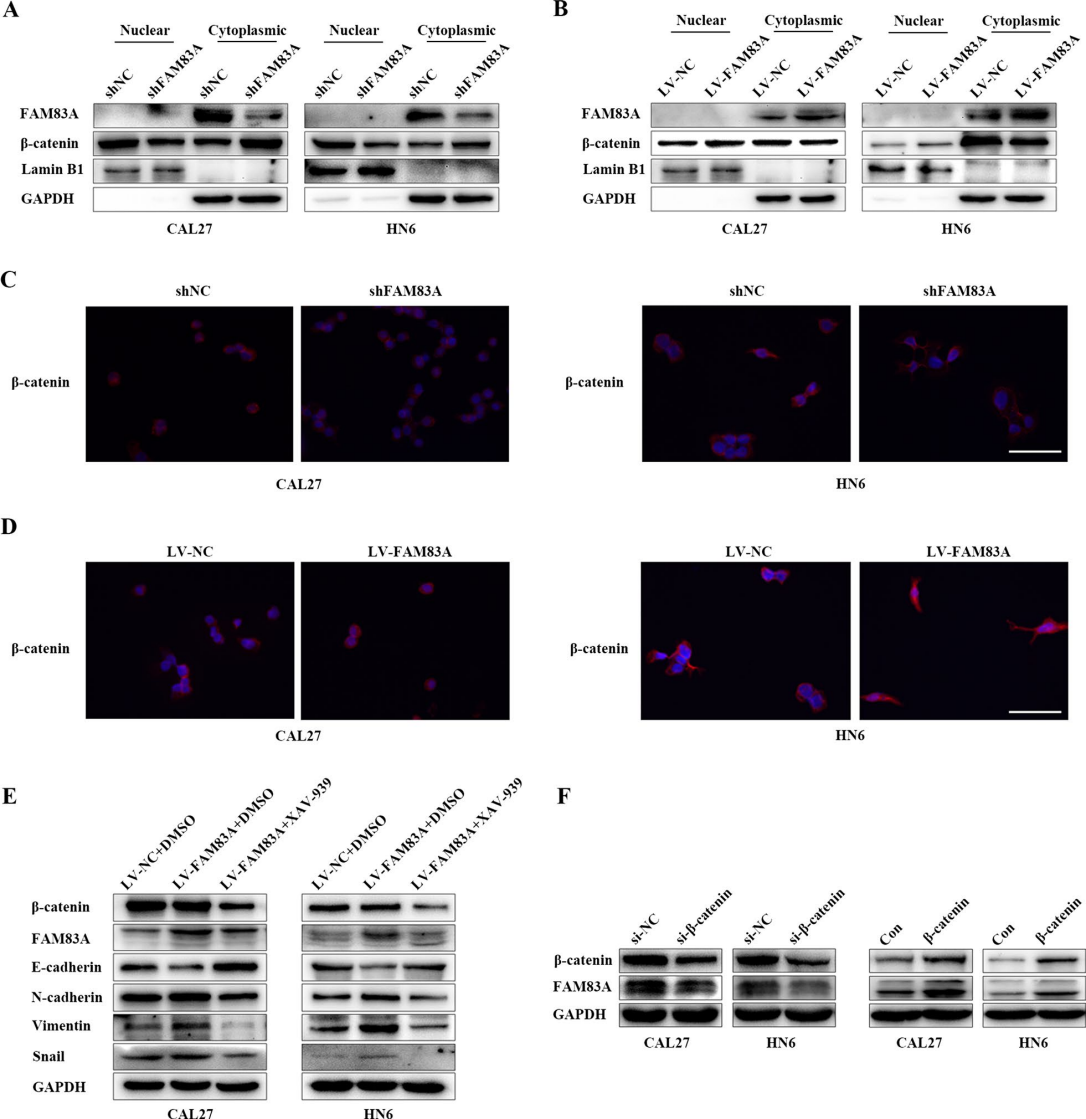
FAM83A promotes EMT through Wnt/β-catenin signaling pathway and β-catenin regulated FAM83A expression in HNSCC cells. **A** Expression of nuclear and cytoplasmic FAM83A and β-catenin protein in CAL27 and HN6 cells after FAM83A silencing. **B** The expressions of nuclear and cytoplasmic FAM83A and β-catenin protein in CAL27 and HN6 cells after FAM83A overexpressed. **C** Immunofluorescence assays of β-catenin in CAL27 and HN6 cells after FAM83A silencing(400 ×). **D** Immunofluorescence assays of β-catenin in CAL27 and HN6 cells after FAM83A overexpressed (400 ×). **E** Protein levels of β-catenin, FAM83A, E-cadherin, N-cadherin, Vimentin, and Snail were determined by western blotting in CAL27 and HN6 cells after being treated with XAV-939. **F** Protein levels of β-catenin and FAM83A were determined by western blotting in CAL27 and HN6 cells after si-β-catenin transfection and plasmid transfection respectively

To further explore the possible mechanism of FAM83A in regulating the proliferation, migration, and invasion of HNSCC, we detected β-catenin changes in nucleus and cytoplasm. Western blotting analysis demonstrated nuclear β-catenin decreased while cytosolic β-catenin increased after FAM83A knockdown in CAL27 and HN6 cells. Meanwhile, β-catenin increased in the nucleus and decreased in the cytoplasm when FAM83A was overexpressed (Fig. 5A and B). Besides, immunofluorescence assay of β-catenin showed lower level of nuclear β-catenin in the FAM83A knockdown groups and higher level of nuclear β-catenin in the FAM83A-overexpressing groups in CAL27 and HN6 cells (Fig. 5C, D). These phenomenon further indicated that FAM83A promoted β-catenin nuclear translocation and activated the Wnt/β-catenin signaling pathway. We treated HNSCC cells with 50 μmol XAV-939 which could suppress exogenous β-catenin expression and we observed the cell viability, migration and invasion ability all decreased after the treatment with XAV-939 in CAL27 and HN6 cells (Additional file 1: Figure S1A–C). At the same time, western blotting assay found that E-cadherin expression was increased, N-cadherin, Vimentin and Snail expression were decreased when CAL27 and HN6 FAM83A-overexpression group cells were treated with XAV-939 (Fig. 5E). Interestingly, the downregulation of β-catenin induced by XAV-939 decreased FAM83A protein expression (Fig. 5E). This suggests that β-catenin could also regulate FAM83A. To further prove it, we treated CAL27 and HN6 cells with small interfering RNA (siRNA) of β-catenin and a plasmid containing the β-catenin cDNA sequence respectively. As shown in Fig. 5F and Additional file 2: Figure S2A–D, β-catenin silencing caused FAM83A downregulation and β-catenin overexpression resulted in FAM83A upregulation both at protein and mRNA level. Taken together, the above findings suggested that FAM83A promotes cell proliferation and metastasis in HNSCC by activating Wnt/β-catenin signaling pathway and there may be a loop feedback between FAM83A and β-catenin.

### FAM83A promoted tumor growth and distant metastasis in vivo

**Fig. 6:**
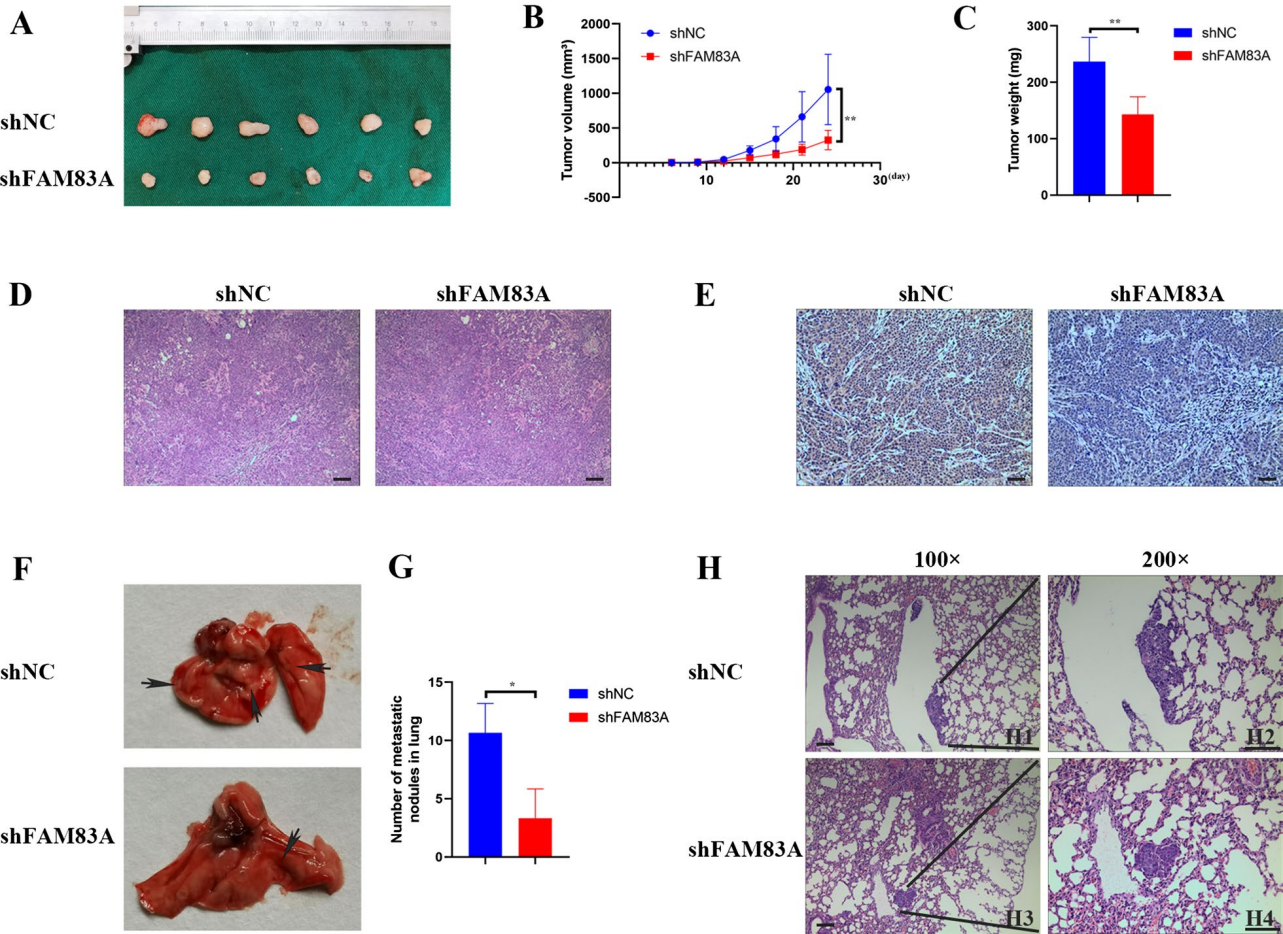
FAM83A knockdown inhibits HNSCC growth and metastasis in nude mice. **A** The shFAM83A cells and control cells were subcutaneously injected into nude mice and general observation of tumor-bearing nude mice injected with cells. **B** Volumes of xenograft tumors in shNC and shFAM83A groups. **C** Tumor tissues in shNC and shFAM83A groups were weighed after 24 days. **D** Representative showings of tumors formed in the nude mice were diagnosed as HNSCC by HE staining (100 ×). **E** Representative photomicrographs of positive staining of FAM83A in shNC groups and negative staining of FAM83A in shFAM83A groups (100 ×). **F**, **G** Representative photographs of lung tissues in mice injected with shFAM83A cells and shNC cells and a summary of the number of lung metastatic nodules in shFAM83A and shNC groups. **H** HE staining of lung tissues in shNC and shFAM83A groups (H1, H3, 100 × ; H2, H4, 200 ×). Data represent the mean ± SD; *p < 0.05, **p < 0.01, ***p < 0.001

To study the function of FAM83A on tumor growth in HNSCC, we used a subcutaneous xenograft tumor model and injected HN6 cells (2 × 10^6^) into the right flank of mice. Compared with the tumors produced by the negative control cells, the tumors separated from mice injected with the FAM83A-knockdown cells were smaller and showed a weaker ability in tumor growth (Fig. 6A). The tumors formed in the negative control group possessed a larger tumor volume and heavier weight than shFAM83A group (Fig. 6B, C). HE staining assay demonstrated the tumors separated from nude mice were HNSCC (Fig. 6D). Besides, IHC assay demonstrated tumors dissected from negative control group had a stronger FAM83A staining compared with the other group (Fig. 6E). Moreover, we used an experimental metastasis assay to explore its function on tumor potential metastasis and injected HN6 cells (1 × 10^6^) into the tail intravenous of nude mice. As described in Fig. 6F and G, mice injected with shFAM83A group cells formed less pulmonary metastases (n = 6, an average of 3 nodules per lung) than shNC group (n = 6, an average of 11 nodules per lung) (Fig. 6F, G). Furthermore, HE staining assays demonstrated that the number of metastatic lesions significantly declined in shFAM83A group mice compared with shNC group (Fig. 6H). These findings suggested that FAM83A promoted tumor growth and metastasis in HNSCC.

## Discussion

HNSCC is a malignant tumor with high mortality rate [1, 2]. The carcinogenesis of HNSCC is complex which involves coefficient functions of multiple feedback loops and cross-talk communications. Different genetic alterations interact with each other and will be taken into consideration to guide the targeted therapy in HNSCC. Previous studies have highlighted that the up-regulated FAM83A often functions as an oncogenic gene in tumorigenesis. However, the function of FAM83A in HNSCC has not been reported and particularly requires further exploration.

In our study, the function and mechanism of FAM83A in HNSCC were profoundly investigated. Firstly, immunohistochemistry in human HNSCC microarrays showed aberrant overexpression of FAM83A in a large number of patients compared with adjacent normal tissues. It is interesting to note that FAM83A positivity was observed as brown membranous and cytoplasmic immunostaining in HNSCC group, consistent with the previous reports [15]. Our results showed that FAM83A expression was positively correlated with advanced tumor size, lymph node metastasis, and clinical tumor stages respectively. The clinical specimens showed that FAM83A may play a key role in the migration and metastasis of HNSCC in the present study. Then, FAM83A expression was noticeably amplified in HNSCC fresh tissues and cells by RT-qPCR and Western Blotting. This was consistent with the results of TCGA database, indicating that HNSCC samples have higher expressions of FAM83A than those in adjacent tissues. Therefore, FAM83A might serve as an oncogene in HNSCC.

The mechanism of FAM83A in promoting HNSCC progression is not clear. To explore the underlying mechanism of FAM83A in HNSCC cells, we constructed stably transfected FAM83A knockdown and overexpressed HNSCC cells. Depletion of FAM83A dramatically inhibited cell proliferation, migration, and invasion in HNSCC cells. Moreover, we found that knockdown of FAM83A expression in HNSCC cells decreased metastatic foci in vivo. FAM83A overexpression exerted the opposite effects. All these results illustrated that the up-regulation of FAM83A could enhance cell proliferation, migration and invasion in HNSCC. Taken together, these data indicate that FAM83A could promote HNSCC progression.

The epithelial to mesenchymal transition (EMT) plays a vital role in cancer metastasis [26, 27]. The down-regulation of epithelium markers such as E-cadherin and the up-regulation of mesenchymal marker such as N-cadherin, Vimentin and Snail are the most common hallmarks of EMT [28]. Prior studies have indicated that initiation of EMT in tumor is frequently observed in the metastasis of HNSCC [29].In our study, HNSCC cells presented fibroblast-like phenotypes, showing a more elongated and spindle-like shape in HNSCC cells after FAM83A overexpression. Also, E-cadherin was increased while N-cadherin, Vimentin, and Snail were inhibited after silencing FAM83A in CAL27 and FADU cell lines, which suggested that the EMT of HNSCC was inhibited. All these data suggested that FAM83A can induce the activation of the EMT-related signaling pathways in HNSCC cells. Therefore, FAM83A might play a vital role in the EMT of HNSCC.

It is suggested that aberrant changes of Wnt/β-catenin signaling pathway are of decisive importance in EMT [30]. Wnt can activate β-catenin at a downstream location in the Wnt/β-catenin signaling [31]. The Wnt/β-catenin signaling pathway can play a key role in cell proliferation, differentiation and regeneration [32]. The upregulation of β-catenin plays an important role in enhancing the invasion and metastasis of several types of cancer by inducing EMT. GSK3β phosphorylates β-catenin and results in the degradation of β-catenin, which inhibits the activity of the Wnt signaling pathway [30–32] Interestingly, it has been reported that the overexpression of FAM83A could interact with the Wnt/β-catenin signaling pathway in pancreatic cancer and lung cancer [6].

To explore the underlying mechanism of FAM83A in HNSCC cells, we examined the effects of FAM83A on the Wnt signaling pathway and EMT. It is reported that c-myc elevated oncogenic activity provided an upregulation of EMT in HNSCC [33].

Our study showed that FAM83A can enhance the expression of active β-catenin and downstream targets of the Wnt pathway, such as cyclin D1 and c-myc. Besides, western blotting assays and immunofluorescence staining assays demonstrated that β-catenin entered into the cell nucleus from the cytoplasm in CAL27 and HN6 FAM83A-upregulated cells, demonstrating the Wnt-pathway-mediated β-catenin activation. These results were further demonstrated by using the β-catenin inhibitor to pharmacologically block the Wnt signaling pathway. In the rescue experiment, the inhibitor, XAV-939, could reverse the promoting effect of FAM83A in HNSCC. It is known that FAM83F and FAM83G can regulate canonical Wnt signaling through an interaction with CK1α [22]. Previous research also proposed that FAM83A can inhibit GSK3β activity and increase the level of active unphosphorylated β-catenin and active β-catenin can transports into the nucleus thus activating the Wnt signaling pathway in lung cancer [23]. Thus, we suspect that FAM83A can similarly lead to aberrant activation of Wnt/β-catenin signaling through its interaction with CK1α and GSK3β in HNSCC. FAM83A can activate β-catenin, leading to the transporting into the nucleus of HNSCC cells. This possibility is under investigation in the lab.

In this study, we found that β-catenin inhibitor could also downregulate the mRNA and protein level of FAM83A in CAL27 and HN6 cells. Furthermore, we found that the overexpression of β-catenin can result in the upregulation of mRNA and protein level of FAM83A. However, there is no β-catenin/TCF/LEF binding site in FAM83A promoter. The fact that the expressions of FAM83A and β-catenin are positively correlated could be explained by the fact that β-catenin can regulate FAM83A through other relative pathways. We think there are possible binding sites of the downstream genes of Wnt/β-catenin in FAM83A promoter. The underlying mechanism needs further investigation.

In general, we presented evidence for the first time that overexpression of FAM83A in HNSCC tissues was positively correlated with advanced tumor size, lymph node metastasis and clinical tumor stages. Besides, the present study is the first to provide evidence that the Wnt-β-catenin-mediated EMT and metastasis by FAM83A in HNSCC, and there may be a potential bi-directional signaling loop between FAM83A and Wnt/β-catenin signaling pathway (Additional file 3: Figure S3). Therefore, understanding the molecular mechanism by which FAM83A activates Wnt signaling pathway in HNSCC will increase our knowledge of the biological basis of HNSCC and may enable the potential strategies for HNSCC. Our research provides a novel target for the early diagnosis and treatment in HNSCC patients.

## Supplementary Information

**Additional file 1: Figure S1**. β-catenin inhibitor named XAV-939 suppresses cell viability, migration and invasion in HNSCC cells. A. The wound-healing assays in CAL27 and HN6 cells after treated with XAV-939 (100×). B. Transwell assays of migration and invasion in CAL27 and HN6 cells after treated with XAV-939 (100×). C. CCK8 assays of the proliferation in CAL27 and HN6 cells after treated with XAV-939. Data represent the mean ± SD; *p< 0.05, **p< 0.01, ***p< 0.001.

**Additional file 2: Figure S2**. β-catenin regulates FAM83A expression in HNSCC cells. A. Gene expressions of β-catenin and FAM83A were measured by RT-PCR in CAL27 cells after si-β-catenin transfection. B. Gene expressions of β-catenin and FAM83A were measured by RT-PCR in HN6 cells after si-β-catenin transfection. C. Gene expressions of β-catenin and FAM83A were measured by RT-PCR in CAL27 cells after β-catenin plasmid transfection. D. Gene expression of β-catenin and FAM83A were measured by RT-PCR in HN6 cells after β-catenin plasmid transfection. Data represent the mean ± SD; *p< 0.05, **p< 0.01, ***p< 0.001.

**Additional file 3: Figure S3**. Hypothesized signaling mechanism involving FAM83A in the development of HNSCC.

## Data Availability

The datasets generated and/or analyzed during the current study are not publicly available but are available from the corresponding author upon reasonable request.
