# Supplementary figures and images for: FAM83A promotes proliferation and metastasis via Wnt/β-catenin signaling in head neck squamous cell carcinoma
Source: J Transl Med. 2021 Oct 12;19:423. doi: 10.1186/s12967-021-03089-6 (PMC8507380; doi:10.1186/s12967-021-03089-6)

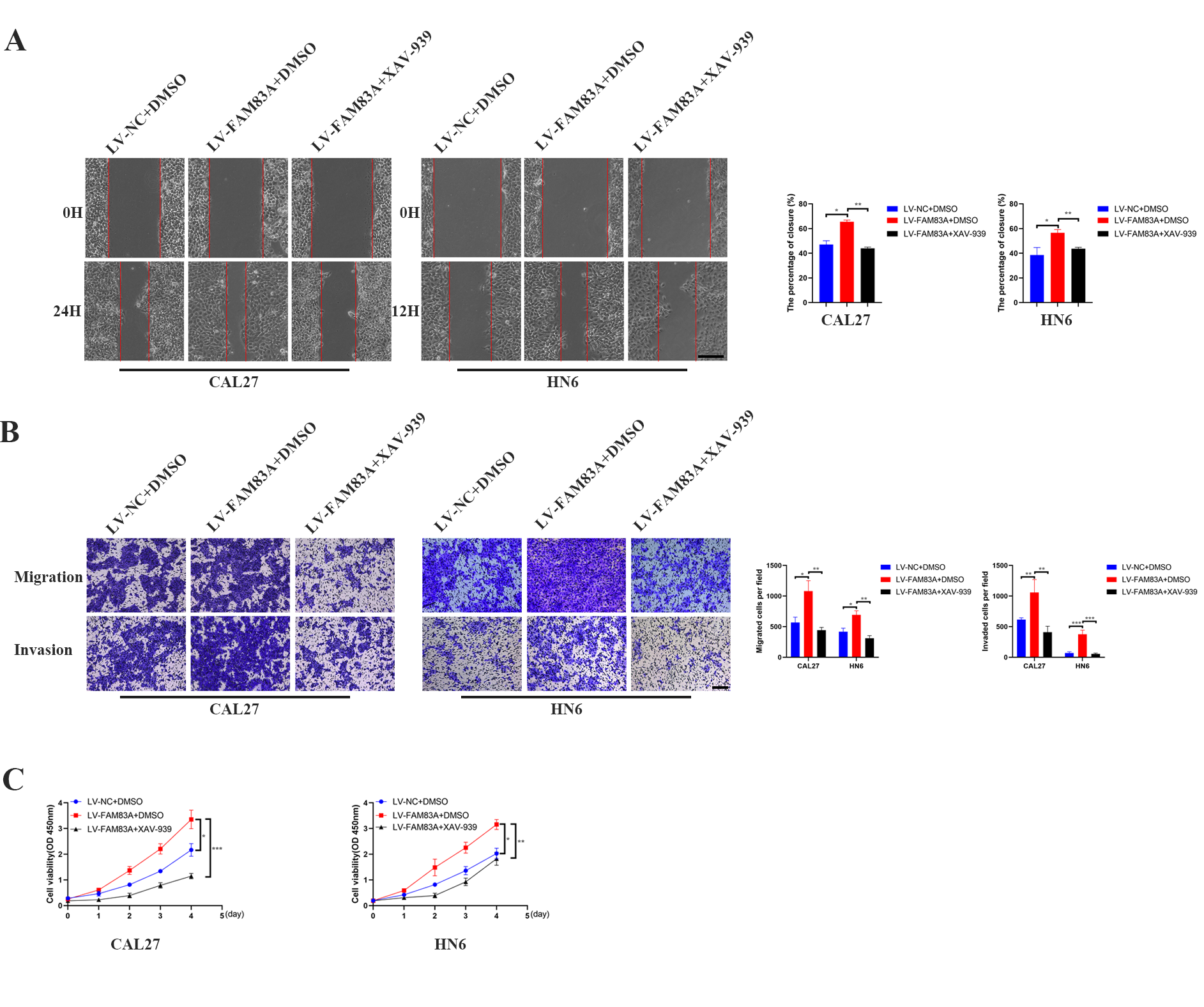

Supplement: Supplementary file 1 — Additional file 1: Figure S1. β-catenin inhibitor named XAV-939 suppresses cell viability, migration and invasion in HNSCC cells. A. The wound-healing assays in CAL27 and HN6 cells after treated with XAV-939 (100×). B. Transwell assays of migration and invasion in CAL27 and HN6 cells after treated with XAV-939 (100×). C. CCK8 assays of the proliferation in CAL27 and HN6 cells after treated with XAV-939. Data represent the mean ± SD; *p< 0.05, **p< 0.01, ***p< 0.001. [file 12967_2021_3089_MOESM1_ESM.tif]

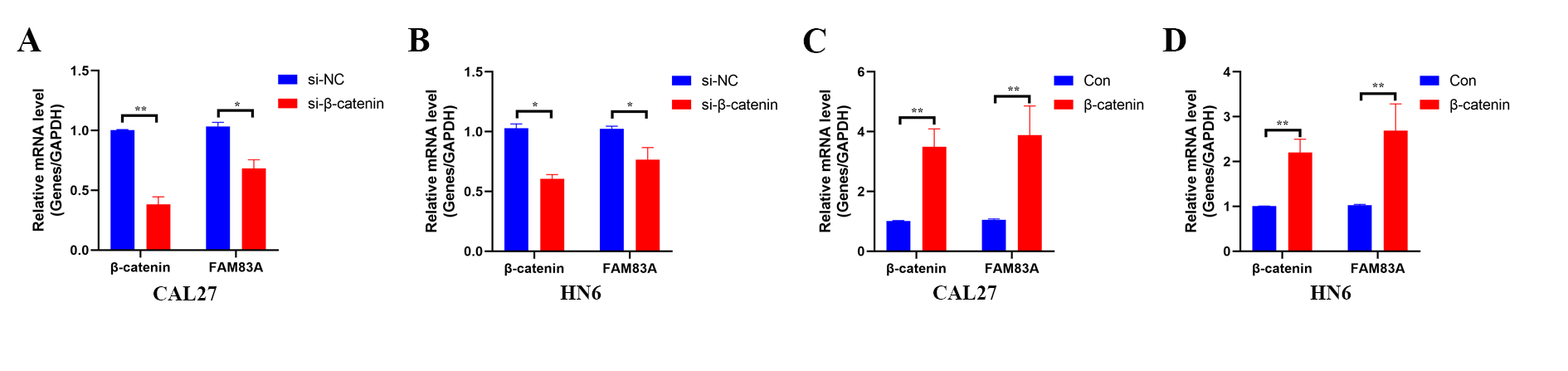

Supplement: Supplementary file 2 — Additional file 2: Figure S2. β-catenin regulates FAM83A expression in HNSCC cells. A. Gene expressions of β-catenin and FAM83A were measured by RT-PCR in CAL27 cells after si-β-catenin transfection. B. Gene expressions of β-catenin and FAM83A were measured by RT-PCR in HN6 cells after si-β-catenin transfection. C. Gene expressions of β-catenin and FAM83A were measured by RT-PCR in CAL27 cells after β-catenin plasmid transfection. D. Gene expression of β-catenin and FAM83A were measured by RT-PCR in HN6 cells after β-catenin plasmid transfection. Data represent the mean ± SD; *p< 0.05, **p< 0.01, ***p< 0.001. [file 12967_2021_3089_MOESM2_ESM.tif]

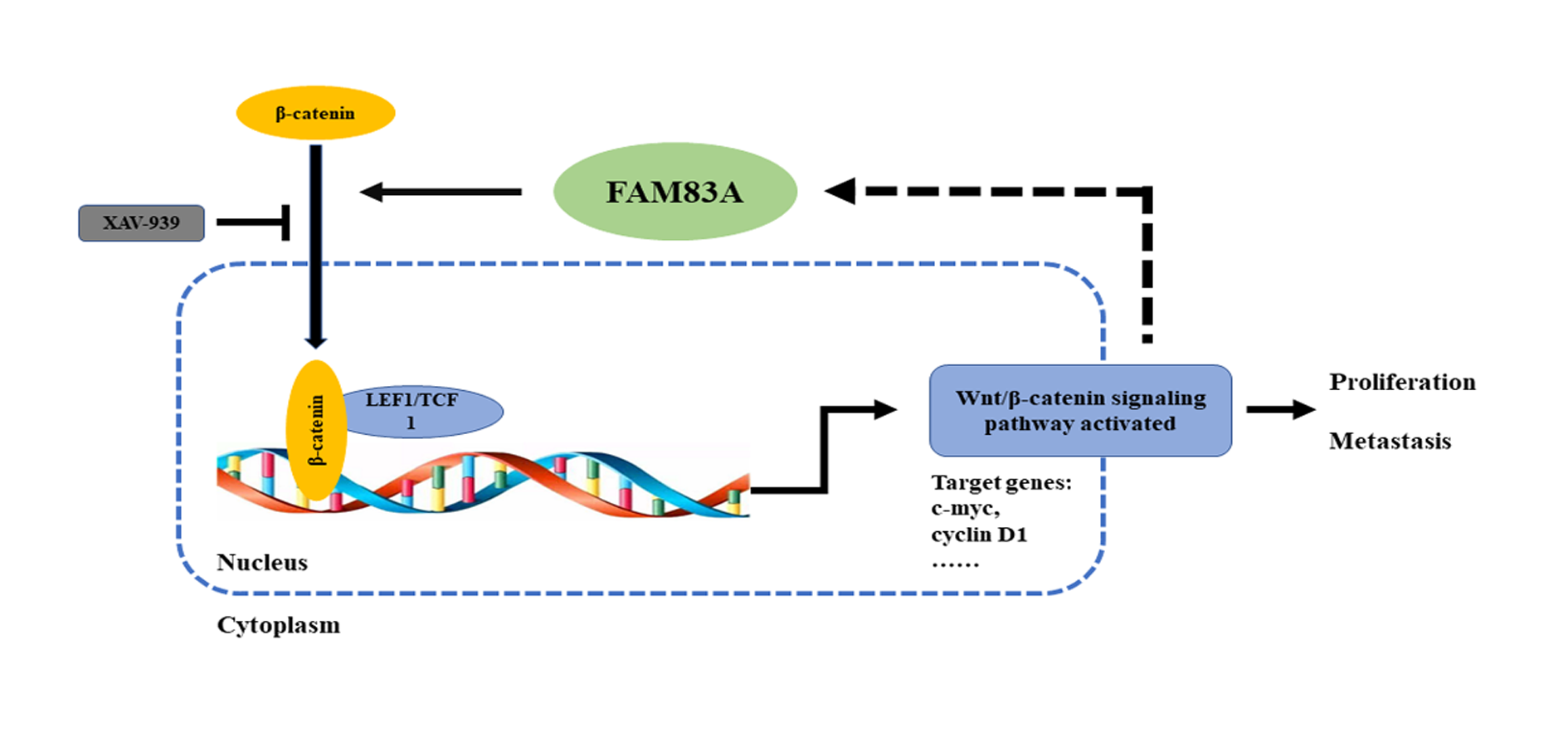

Supplement: Supplementary file 3 — Additional file 3: Figure S3. Hypothesized signaling mechanism involving FAM83A in the development of HNSCC. [file 12967_2021_3089_MOESM3_ESM.tif]
